# The quality of pharmacist-led community warfarin management across 2 provinces in Canada: A cross-sectional observational study

**DOI:** 10.1177/17151635241228228

**Published:** 2024-02-05

**Authors:** Laura Morrison, Jeff Nagge

**Affiliations:** School of Pharmacy, University of Waterloo; School of Pharmacy, University of Waterloo; Michael G. Degroote School of Medicine, Department of Family Medicine, McMaster University

## Abstract

**Background:**

Guidelines for anticoagulation management services recommend personnel be specially trained in warfarin management and suggest using tools such as decision-support software. To date, there have been no Canadian studies documenting the quality of warfarin management using a similar guideline recommended approach.

**Methods:**

A cross-sectional, retrospective observational study was conducted to measure the quality of pharmacist-led warfarin management using point-of-care international normalized ratio (INR) testing and decision-support software in various ambulatory settings in Canada. Settings included 4 family health teams in Ontario and 40 community pharmacies across Nova Scotia. Quality was measured using time in therapeutic range (TTR) and was reported in 3 manners: mean TTR, median TTR and time-weighted mean TTR.

**Results:**

The primary outcome included 963 patients. The combined mean and median TTR for the 2019 Ontario family health teams and Nova Scotia pharmacies was 74.2% and 77.3% (interquartile range 64%-87.9%), respectively. The time-weighted mean TTR was 76.3%.

**Discussion:**

To the best of our knowledge, the TTR achieved by this model of care is the highest reported in Canadian general practice. Since Thrombosis Canada defines good-quality warfarin management as a TTR of 60% or greater, and many studies have reported an association between higher TTR values and lower rates of thrombosis and hemorrhage, this model of care may have significant benefits for patients.

**Conclusion:**

This study demonstrates the high quality of anticoagulation management provided by specially trained pharmacists using point-of-care INR testing and decision-support software. These results support expanded access to this service for all Canadians. *Can Pharm J (Ott)* 2024;157:xx–xx.

## Introduction

Approximately 200,000 Canadians have atrial fibrillation. Many are prescribed oral anticoagulant therapy (OAT) to reduce the risk of stroke.^1^ Warfarin was the OAT used by most Canadian patients prior to 2010, when oral factor IIa inhibitor dabigatran was introduced. Three oral anti–factor Xa inhibitors (apixaban, rivaroxaban and edoxaban) followed, all sharing the advantage of not requiring INR monitoring. These newer oral anticoagulants are often referred to as direct-acting oral anticoagulants (DOACs). Although DOACs are now recommended by guidelines as the preferred OAT for most, there are still patients for whom warfarin is more appropriate (e.g., artificial heart valves, poor adherence, significant renal impairment or patient preference).^2^

The quality of warfarin management can be evaluated using a metric called “time in therapeutic range” (TTR).^3^ A 2014 meta-analysis analyzed quality of management along with outcomes.^4^ It found an association between higher TTR values and lower rates of thrombosis and hemorrhage, with 57% of thromboembolic events occurring in patients with an international normalized ratio (INR) <2.0 and 42% of hemorrhagic events occurring in patients with an INR >3.0.^4^ While Thrombosis Canada defines good-quality warfarin management as a TTR of 60% or greater, others suggest <65% is low quality.^5,6^ A few studies have reported the quality of warfarin management in various Canadian practices, with TTR ranging from 55.4% to 67.8%. Most patients in these studies had their warfarin therapy managed by family physicians, and none reported using point-of-care testing^7-10^ (Table 1).

**Table 1:** Comparison of Canadian studies reporting on TTR

Reference, date	Study details	Provider	TTR	Comparison to current study
Wilson et al., *CMAJ* 2003	Patients taking wafarin for all indications Time censored for planned interruptions of warfarin Three sites (2 in Ontario, 1 in Nova Scotia)	112 patients managed by multidisciplinary anticoaguation team 109 managed by family physician	*Mean TTR = 63% *Mean TTR = 59%	Mean TTR = 74.2%
Gateman et al., *Can Fam Physician* 2017	Patients taking warfarin for nonvalvular atrial fibrillation One Ontario family health network	150 patients managed by nurse-administered warfarin anticoagulation protocol	Time-weighted mean TTR = 58.8%	Time-weighted mean TTR = 76.3%
Saber et al., *Can J Cardiol* 2012	Patients taking warfarin with therapeutic range 2-3 Those with 5 or fewer INR values and/or more than 6 months between test dates excluded Across British Columbia	50,404 patients with community laboratory data	Mean TTR = 55.4%	Mean TTR = 74.2%
Liu et al., *Can Fam Physician* 2019	Patients taking warfarin for atrial fibrillation or thrombosis At least 7 INRs, with first 5 for each patient removed Seven Canadian provinces	5556 patients managed in primary care	**Median TTR = 67.8%	Median TTR = 77.3%

INR, international normalized ratio; TTR, time in therapeutic range.

* Results for TTR using standard therapeutic range.

** Results for TTR using range 2 to 3.

Guidelines for anticoagulation management services recommend personnel be specially trained and suggest using tools such as decision-support software.^11^ The benefits of this approach are evident in a New Zealand study of 693 patients whose warfarin was managed by trained community pharmacists using point-of-care testing and computerized decision-support software. This resulted in a TTR of 78.6%.^12^ To date, there have been no Canadian studies documenting the quality of warfarin management using a similar guideline-recommended approach.

This study measures the quality of specially trained pharmacist-led warfarin management using point-of-care INR testing and decision-support software in various ambulatory settings in Canada.

**Knowledge into Practice**

- Guidelines for anticoagulation management services recommend personnel be trained in warfarin management and suggest using tools such as decision support software.
- To date, there have been no Canadian studies documenting the quality of warfarin management using a similar guideline-recommended approach.
- This study demonstrates the high quality of anticoagulation management provided by specially trained pharmacists using point-of-care INR testing and decision-support software with a time-weighted mean TTR of 76.3%, the highest ever reported in Canadian general practice.
- These results support expanded access to this service for all Canadians.

**Mise En Pratique Des Connaissances**

- Les lignes directrices en matière de services de prise en charge de l’anticoagulation recommandent que le personnel soit formé à la prise en charge de la warfarine et suggèrent d’utiliser des outils tels que des logiciels d’aide à la décision.
- À ce jour, aucune étude canadienne n’a documenté la qualité de la prise en charge de la warfarine à l’aide d’une approche semblable recommandée dans les lignes directrices.
- Cette étude démontre la grande qualité de la prise en charge de l’anticoagulation par des pharmaciens spécialement formés qui utilisent un logiciel d’analyse du RIN et d’aide à la décision hors laboratoire avec un TTR moyen pondéré en fonction du temps de 76,3 %, soit le taux le plus élevé jamais enregistré en pratique générale au Canada.
- Ces résultats favorisent un accès élargi à ce service pour tous les Canadiens.

## Methods

### Study design and setting

The settings included 4 family health teams (FHT) in Ontario (New Vision FHT, Kitchener; Queen’s FHT, Belleville; City of Kawartha Lakes FHT, Lindsay; and Niagara North FHT, St. Catherine’s) and 40 community pharmacies across Nova Scotia. Sites were selected because all were primarily pharmacist led. Pharmacists had delegated authority to adjust warfarin doses to maintain patients within therapeutic range. Pharmacists completed standardized training with a certificate program in the management of oral anticoagulation therapy offered by the University of Waterloo and used INROnline as a computerized decision-support software. INRs were determined using point-of-care testing (CoaguChek XS, CoaguChek XS Pro or Coaguchek INRange, Roche). Three of the FHTs had nurses involved in their programs.

At each test encounter, patients are asked if they missed a warfarin dose, started new medications, were hospitalized or experienced any bleeding or bruising. Answers to these questions, along with INR value and warfarin dosing recommendation, are entered into INROnline and stored within their servers. INROnline uses the stored information to calculate a dose and date for the next test. Clinicians had the ability to override the recommendation.

### Participants

Patients had to have a diagnosis of nonvalvular atrial fibrillation and be taking warfarin therapy managed primarily by specially trained pharmacists using point-of-care INR testing and decision support at 1 of the 4 FHTs in Ontario or community pharmacies in Nova Scotia.

### Outcomes and study time frame

The primary outcome was to compare TTR (Rosendaal method)^3^ across all sites from January 1, 2019, to December 31, 2019, to the TTR defined by Thrombosis Canada as good control. This time frame was chosen because all sites had data available for this window.

There were 2 prespecified secondary outcomes. First, a trend analysis across 3 time periods using only data from Ontario FHTs: January 1, 2013, to December 31, 2013; January 1, 2017, to December 31, 2017; and January 1, 2021, to December 31, 2021. We used only Ontario data for this analysis because the Nova Scotia sites were not operating until 2018. We aimed to determine if changes to warfarin-user demographics and complexities resulting from increased use of DOACs affected the quality of warfarin management.

Second, data between January 1, 2019, and December 31, 2019, were analyzed to compare the TTR between the 4 Ontario FHT sites and the Nova Scotia community pharmacies.

### Data

Anonymous data were provided in kind by INROnline. Data were received in an Excel spreadsheet from Firecrest Systems, an information technology company contracted by INROnline.

**Figure 1:**
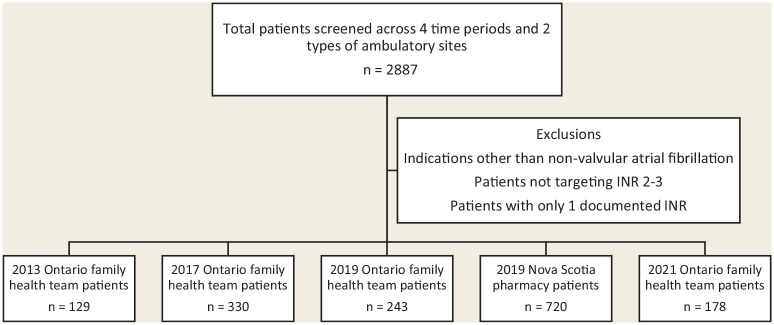
Flow diagram of patients screened *Screening was done for Ontario family health teams between January 1, 2013, and December 31, 2013; January 1, 2017, and December 31, 2017; January 1, 2019, and December 31, 2019; January 1, 2021, and December 31, 2021, as well as for Nova Scotia pharmacies between January 1, 2019, and December 31, 2019.

The data included all INR results reported for the sites and time periods listed. First, data were reviewed to include only patients with diagnosis of nonvalvular atrial fibrillation targeting an INR of 2 to 3 to ensure similarity of comparisons. INRs of 0 were removed, as this is physiologically impossible. Patients with only 1 INR recorded were removed since the Rosendaal method requires 2 consecutive INR determinations to calculate TTR (Figure 1).

TTR was then calculated for each patient using Rosendaal’s method. These calculations were performed by 1 investigator using a formatted Microsoft Excel spreadsheet and independently validated by another investigator. To permit comparison with previous Canadian studies, the TTR was reported in 3 manners: mean TTR (i.e., average of the individual TTRs at a specific study site), median clinic TTR and time-weighted mean TTR (i.e., overall TTR at a specific study site calculated as the overall number of days patients were within their therapeutic range divided by the number of patient-days within the time frame).

### Statistical analysis

For the primary objective, descriptive statistics were used to analyze the data using means, medians (interquartile range) and time-weighted means.

For the secondary objective trend analysis, a single-factor analysis of variance (ANOVA) test was performed followed by multiple comparisons using Tukey’s post hoc test to compare means across the 3 time periods. Descriptive statistics were used to analyze the data using means, medians (interquartile range) and time-weighted means.

For the secondary objective comparing the types of ambulatory practice sites, *f*-test followed by *t*-test for unequal variance was performed using mean values.

### Ethics approval

Ethics approval was secured from the Office of Research Ethics at the University of Waterloo. A copy of the ethics clearance and study protocol was provided to study sites.

## Results

The complete data set included 2887 patients with 2957 indications for warfarin. Atrial fibrillation accounted for approximately 70% of indications. Other indications included deep vein thrombosis, pulmonary embolus, mechanical heart valve, post–myocardial infarction, tissue heart valve, transient ischemic attack, mural thrombus and other.

The primary outcome included 963 patients with an average of 15 INR tests per patient in 2019. The mean and median duration of warfarin therapy included in the 2019 data was 285.8 days and 334 days (interquartile range 287-343.9 days), respectively. The average patient age was approximately 78 years, and 57% were male. Ontario FHT data contributed 243 patients, and Nova Scotia community pharmacy data included 720 patients. The overall mean and median TTR for the 2019 Ontario FHTs and Nova Scotia pharmacies was 74.2% and 77.3% (interquartile range 64%-87.9%), respectively. The time-weighted mean TTR was calculated as 76.3% (Table 2).

**Table 2:** Patient characteristics and data from 2019

	Ontario family health teams 2019	Nova Scotia pharmacies 2019	Total 2019
Number of patients (% male)	243 (55)	720 (57)	963 (57)
Mean age, years (range)	79 (44-104)	77 (44-100)	78 (44-104)
Mean number of tests per patient (range)	13.7 (2-35)	15.4 (2-50)	15 (2-50)
Mean number of days of warfarin therapy included in data (range)	274.4 (2-359.6)	289.6 (3-362)	285.8 (2-362)
Median number of days of warfarin therapy included in data (interquartile range)	329 (226.5-342.9)	334.9 (302.6-344.1)	334 (287-343.9)
Mean TTR (%)	73	74.6	74.2
Time-weighted mean TTR (%)	76.5	76.3	76.3
Median TTR (interquartile range, %)	77.5 (63.7-86)	77.2 (64-88.2)	77.3 (64-87.9)

TTR, time in therapeutic range.

When analyzing the time trend in mean TTR values across 3 time periods, a statistically significant between-group difference was found (*p* = 0.02, single-factor ANOVA; Table 3). Multiple comparisons by Tukey’s post hoc test were performed to further analyze the differences. Marginally significant differences were found when comparing the mean TTR in 2013 to 2017 (*p* = 0.04) and between 2013 and 2021 (*p* = 0.02). The comparison in mean TTR between 2017 and 2021 was not significant (*p* = 0.80). We were unable to compare patient-reported bleeding and hospitalizations due to inconsistent reporting and insufficient validation of this data field.

**Table 3:** Patient characteristics and data from Ontario family health teams across 3 time periods

	Ontario family health teams 2013	Ontario family health teams 2017	Ontario family health teams 2021
Number of patients (% male)	129 (57)	330 (56)	178 (58)
Mean age, years (range)	77 (42-97)	78 (32-102)	80 (52-106)
Mean number of tests per patient (range)	8.4 (2-24)	13.7 (2-37)	13.6 (2-53)
Mean number of days of warfarin therapy included in data (range)	128.2 (6.8-331.6)	274.3 (6-359.1)	269.1 (7.8-355.1)
Median number of days of warfarin therapy included in data (interquartile range)	132.3 (47.4-186.3)	327.1 (235.7-342.4)	327.3 (233.5-339.1)
Mean TTR (%)	68.4	74	75.3
Time-weighted mean TTR (%)	70.6	77.5	76.7
Median TTR (interquartile range, %)	71.8 (53.5-87.4)	79.8 (64.2-89.7)	79.4 (63.7-88.6)

TTR, time in therapeutic range.

There was no difference in the quality of warfarin management between the types of ambulatory sites. The mean 2019 TTR was 73% and 74.6% for Ontario FHTs and Nova Scotia community pharmacies, respectively (*p* = 0.28, *t*-test for unequal variance). Similar results were obtained when comparing time-weighted mean TTRs (76.5% for Ontario FHTs and 76.3% for Nova Scotia community pharmacies; Table 2).

## Discussion

To the best of our knowledge, the TTR achieved by this model of care is the highest reported in Canadian general practice. When comparing the TTR across studies, it is important to consider several factors. First, studies may report the mean TTR, median TTR or time-weighted TTR. For example, one trial using community laboratory data from British Columbia reported a mean TTR of 55.4%.^9^ Another study investigating TTR for 150 patients with atrial fibrillation managed by a nurse-administered warfarin dosing protocol found a time-weighted TTR of 58.8%.^8^ The mean and time-weighted mean TTRs reported for the primary outcome in our study were nearly 20% higher than either of these results (74.2% and 76.3%, respectively). Second, some studies report on an expanded therapeutic range. The primary outcome measure for a trial of 221 patients randomized to warfarin management by either their family physician or anticoagulation clinic was TTR ±0.2 INR units, referred to as “expanded therapeutic range.”^7^ The TTR for this expanded range was reported as 76% and 82%, respectively. When the analysis was restricted to the standard therapeutic range, the TTR was 59% and 63%, respectively.^7^ Third, some studies will censor INR values for periods of time. For example, the randomized trial discussed previously censored INR values from the day warfarin was discontinued for a planned interruption until 5 days after restarting.^7^ Another study investigating the TTR for patients managed across 7 Canadian provinces using data from the Canadian Primary Care Sentinel Surveillance Network reported a median TTR of 67.8% for patients with known atrial fibrillation or venous thromboembolism.^10^ This study required patients have at least 7 INRs to be included, and the first 5 for each patient were removed. Despite not using any of these adjustments that would artificially improve TTR, the median TTR in our study was nearly 10% higher (77.3%).

The increased TTR found that using the model of care described in our study may have significant benefits for patients. A systematic review examining the relationship between INR control and risk of adverse clinical events found that a 7% improvement in TTR was associated with 1 fewer major hemorrhage per 100 patient-years and a 12% improvement with 1 less thromboembolic event per 100 patient-years.^13^

Adding further support to this model of warfarin monitoring is the similarity in the TTR we report to that of one in New Zealand that included the same factors of standardized training, computerized decision support and point-of-care INR testing.^12^ The New Zealand study reported a median duration of follow-up of 197 days and a median of 3.4 INR tests per month. The authors followed a protocol in which testing initially occurred once a week, with the interval between tests gradually extending to a maximum of 4 weeks. They comment that the median interval between tests rose when comparing the first 3 months of enrollment to months 4 to 6. Our study’s primary outcome had a median duration of follow-up of 334 days and a median of approximately 1.4 tests per month. We suspect that the difference in number of tests per month is due to more frequent testing performed initially in the New Zealand study, whereas our study did not follow such protocol.

Our secondary outcome found a marginally significant trend of improving TTR over the time periods. If TTR truly was increasing, it might be related to increased clinician experience and comfort over time. Also, patients with atrial fibrillation receiving public drug coverage in Ontario and Nova Scotia must first try warfarin therapy, unless contraindicated or not possible due to inability to monitor. This may channel patients with poor TTR to DOAC therapy, while those remaining on warfarin may have more stable INR control. While this is a logical explanation, a study including more than 18,000 patients suggested that past INR stability does not predict future INR control.^14^

Our data suggested no difference between the quality of warfarin management between types of ambulatory practice sites. This provides support that with standardized training, pharmacist-led warfarin management using point-of-care INR testing and decision-support software can be effective regardless of the setting. Whether the pharmacist is embedded within a multidisciplinary team with readily available support from other clinicians or practising in a community pharmacy, the quality appears to be similar.

## Limitations

There are several limitations to note. First, this is a retrospective, observational study relying on INR values obtained from electronic documentation. We believe it is unlikely that INR values would have been missed or entered incorrectly, as clinicians were trained and would have noticed an unreasonable warfarin dose recommendation. In addition, 2 researchers cleaned the data and calculated TTR independently. Second, our data source (INROnline) provided limited clinical and demographic information about the patients enrolled. This impairs the ability to directly compare the results to those reported in other studies. Third, we did not have access to clinical outcomes and instead relied on the surrogate measure of TTR. Finally, the sample size for our trend analysis was limited, and *p*-values were marginally significant, so results should be considered hypothesis-generating only.

## Conclusion

This study demonstrates the high quality of anticoagulation management provided by specially trained pharmacists using point-of-care INR testing and decision-support software in various ambulatory settings across 2 provinces. These results support expanded access to this service for all Canadians. ■
